# The effects of environmental hypoxia on substrate utilisation during exercise: a meta-analysis

**DOI:** 10.1186/s12970-019-0277-8

**Published:** 2019-02-27

**Authors:** Alex Griffiths, Oliver M. Shannon, Jamie Matu, Roderick King, Kevin Deighton, John P. O’Hara

**Affiliations:** 1 Institute for Sport, Physical Activity and Leisure, Leeds Beckett University, Leeds, LS6 3QS UK; 2 Human Nutrition Research Centre, Institute of Cellular Medicine, Newcastle University, Leech Building, Framlington Place, Newcastle Upon Tyne, NE2 4HH UK; 3 Leeds Institute of Rheumatic and Musculoskeletal Medicine, 2nd floor Chapel Allerton Hospital, Chapeltown Road, Leeds, LS7 4SA UK

**Keywords:** Altitude, Exercise, Substrate, Carbohydrate, Fat, Oxidation, Systematic review

## Abstract

**Background:**

A better understanding of hypoxia-induced changes in substrate utilisation can facilitate the development of nutritional strategies for mountaineers, military personnel and athletes during exposure to altitude. However, reported metabolic responses are currently divergent. As such, this systematic review and meta-analysis aims to determine the changes in substrate utilisation during exercise in hypoxia compared with normoxia and identify study characteristics responsible for the heterogeneity in findings.

**Methods:**

A total of six databases (PubMed, the Cochrane Library, MEDLINE, SPORTDiscus, PsychINFO, and CINAHL via EBSCO*host*) were searched for published original studies, conference proceedings, abstracts, dissertations and theses. Studies were included if they evaluated respiratory exchange ratio (RER) and/or carbohydrate or fat oxidation during steady state exercise matched for relative intensities in normoxia and hypoxia (normobaric or hypobaric). A random-effects meta-analysis was performed on outcome variables. Meta-regression analysis was performed to investigate potential sources of heterogeneity.

**Results:**

In total, 18 studies were included in the meta-analysis. There was no significant change in RER during exercise matched for relative exercise intensities in hypoxia, compared with normoxia (mean difference: 0.01, 95% CI: -0.02 to 0.05; *n* = 31, *p* = 0.45). Meta-regression analysis suggests that consumption of a pre-exercise meal (*p* < 0.01) and a higher exercise intensity (*p* = 0.04) when exposed to hypoxia may increase carbohydrate oxidation compared with normoxia.

**Conclusions:**

Exposure to hypoxia did not induce a consistent change in the relative contribution of carbohydrate or fat to the total energy yield during exercise matched for relative intensities, compared with normoxia. The direction of these responses appears to be mediated by the consumption of a pre-exercise meal and exercise intensity.

**Electronic supplementary material:**

The online version of this article (10.1186/s12970-019-0277-8) contains supplementary material, which is available to authorized users.

## Background

An increasing number of people ascend to altitude each year for recreational, occupational, and sporting purposes [1]. The hypoxic exposure experienced at altitude is known to cause a curvilinear impairment in endurance performance with increasing levels of hypoxia [2]. However, the changes in substrate utilisation associated with these decrements in performance are currently unclear, with some authors reporting an increased contribution of carbohydrate to the total energy yield [3, 4], and others demonstrating an increased contribution of fat oxidation [5, 6]. Developing a better understanding of these changes in substrate utilisation in hypoxia is vital in designing dietary interventions to maintain and/or improve performance in such conditions.

Exposure to hypoxic environments may alter substrate utilisation through multifarious mechanisms. It is purported that the mechanism responsible for increased carbohydrate dependency in hypoxia is mediated by the sympathetic nervous system, via the secretion of epinephrine and norepinephrine, stimulating glycogenolysis and gluconeogenesis [3, 7]. Alternative evidence suggests that increases in the transcription factor hypoxic-inducible factor 1 alpha (HIF-1α) may be responsible for the increases in fat oxidation observed by some in hypoxia, via upregulation of the fatty acid-activated transcription factor peroxisome proliferator-activated receptor alpha (PPARα) [8]. Albeit in rats, PPARα has been demonstrated to deactivate pyruvate dehydrogenase [9], inhibiting the conversion of pyruvate to acetyl-coA and therefore enabling greater fat flux for oxidation [10].

A myriad of factors has been suggested to influence the interaction between hypoxic exposure and substrate utilisation. These include, but are not limited to: characteristics of hypoxic exposure (severity, duration and type) [3], nutritional status of participants (fasted or fed before exercise/exogenous supplementation during exercise) [4, 5] and sex of participants [11]. In relation to hypoxic exposure, hypobaric hypoxia (HH) has been suggested to elicit more severe physiological responses (greater hypoxemia and lower arterial oxygen saturation) compared with normobaric hypoxia (NH) [12]. Although this is contested in the literature [13, 14] it seems plausible that these physiological differences may induce a greater reliance on carbohydrate oxidation to achieve a higher yield of ATP per unit of oxygen consumption, compared with fat oxidation [15]. This theory may also be applied to the effect of altitude severity on subsequent substrate oxidation. Further, metabolic responses may be different between sex, with females demonstrating a greater relative utilisation of fat oxidation [11, 16]. This may be attributable to a number of factors, with females demonstrating a greater relative fat mass [17] and intramuscular triglyceride stores [18], as well as better mobilisation of free fatty acid (FFA) from subcutaneous adipose tissue [19]. It has also been suggested that this propensity for fat oxidation may be mediated by the steroid hormones estrogen (predominantly 17 β-estradiol) and progesterone [20, 21]. Regarding nutritional status of participants, equivocal metabolic findings have been observed in response to carbohydrate supplementation during exercise in hypoxia, compared with normoxia [4, 5], which demonstrates the limited current understanding of the interaction between dietary interventions and hypoxic exposure.

To gain a clear understanding of changes in substrate utilisation during exercise in hypoxia compared with normoxia, a systematic evaluation is required to explain the equivocal results of previous studies. Due to the greater exercise-induced physiological stress experienced when performing a matched absolute workload under hypoxic conditions [22], this meta-analysis focuses solely on exercise matched to relative intensities. The aim of this meta-analysis was to identify the study characteristics responsible for heterogeneity between findings, using subgroup analyses and meta-regression.

## Methods

The current systematic review and meta-analysis was performed in accordance with the Preferred Reporting Items for Systematic Review and Meta-analyses (PRISMA) guidelines [23].

### Literature search

A literature search was conducted using the electronic bibliographic databases PubMed and the Cochrane Library, as well as searching MEDLINE, SPORTDiscus, PsychINFO, and CINAHL via EBSCO*host*. The initial search of titles, abstracts and keywords was conducted on 15th November 2016 using terms related to ‘exercise’, ‘hypoxia’, ‘substrate’ and ‘oxidation’. A final search was conducted on 5th June 2018. The specific keywords and full search strategy can be found in Additional file 1. The reference lists of all included studies and relevant review articles were screened for possible inclusion. No language restrictions were applied and in the case of studies available only as an abstract, authors were contacted for the full dataset.

### Inclusion criteria

Included studies were required to meet the following criteria: participants in the study were between the ages of 18 and 65 years, not pregnant, non-smokers, with no history of diabetes, gastrointestinal, inflammatory, metabolic, cardiovascular, neurological or psychological disease. In order to minimise potential publication bias, studies published in peer reviewed journals, conference proceedings, theses or dissertations were eligible for inclusion.

All studies were required to measure RER and/or carbohydrate or fat oxidation. These measures were required to be quantified during exercise matched for relative intensities in hypoxic and normoxic environments. Hypoxic exposure was defined as terrestrial altitude via geographical location (TA) or simulated altitude (NH or HH) via a hypoxic tent, hypoxic chamber or breathing mask. Exposures were required to be > 1500 m or a simulated equivalent (i.e., low altitude or higher) [24]. All participants within selected studies had not been exposed to > 1500 m (or a simulated equivalent) within the previous 3 months. Normoxic trials were required to provide a viable within-participant control (i.e. equivalent measure(s) quantified in the same participants as a separate trial in normoxic conditions). The exercise was required to be > 5 min in duration to achieve steady-state values at a fixed exercise intensity [25].

Two researchers (AG and OS) independently assessed studies for inclusion and later compared notes to reach a mutual consensus. Disagreements about the eligibility of any particular studies were resolved by a third reviewer (KD). Potential studies that could not be excluded based on their title or abstract were retrieved in full-text and reviewed against the inclusion/exclusion criteria independently by two researchers (AG and OS) with a third researcher (KD) used to settle any disputes. In total, 18 studies met the inclusion criteria and were included in this meta-analysis.

### Abstraction of data

Data were extracted independently by two researchers (AG and OS) into a standardised spreadsheet, which included (i) characteristics of articles valid for review; (ii) the Cochrane Collaboration’s tool for assessing risk of bias, and (iii) outcome data suitable for analysis based on mean, standard deviation (SD) and sample size. Further data was extracted regarding participant characteristics, acclimatisation status, nutritional manipulations, exercise intensities and duration, exercise mode, and severity and duration of hypoxic exposure. In studies which employed multiple exercise intensities, each respective intensity was directly compared with the equivalent intensity in the alternate condition.

In studies which reported outcome variables across numerous time points during exercise, values were averaged to calculate the mean. In addition, SD values were averaged using the following formula:

$$ \frac{{\mathrm{n}}^1\left(\mathrm{S}{1}^2+\mathrm{D}{1}^2\right)+{\mathrm{n}}^2\left(\mathrm{S}{2}^2+\mathrm{D}{2}^2\right)\dots }{\left({\mathrm{n}}^1+{\mathrm{n}}^2\right)\dots } $$

where:

n^1^ = sample size of group 1

n^2^ = sample size of group 2

S1 = SD of group 1

S2 = SD of group 2

D1 = mean of group 1 – mean of total group

D2 = mean of group 2 – mean of total group

… denotes inclusion of further data points if required

Absolute substrate oxidation data was converted to g·min^− 1^. Thus, values expressed as total grams oxidised throughout exercise were divided by the number of minutes the variable was measured. Values expressed in mg·kcal·min^− 1^ were multiplied by 1000 to convert to g·kcal·min^− 1^, and then multiplied by kcal values provided in the relevant paper for the conversion to g·min^− 1^. In addition, carbohydrate oxidation data provided in mmol·min^− 1^ were divided by 1000 and then multiplied by the molar mass of glucose (180.1559 g/mol). Where values were presented as figures, these were digitized using graph digitizer software (DigitizeIt, Germany) and the means and SD were measured manually at the pixel level to the scale provided on the figure.

#### Assessment of risk of bias in included studies

Two independent reviewers (AG and OS) used The Cochrane Collaboration’s tool for assessing risk of bias [26] to determine the risk of bias in each study. Each study was assessed in the following six domains: sequence generation, allocation concealment, blinding of participants, personnel and outcome assessors, incomplete outcome data, selective outcome reporting and other sources of bias (e.g. has been claimed to have been fraudulent). A judgement was made on each of the domains by the two independent researchers as to whether they were ‘high risk ‘or ‘low risk’. When insufficient detail was reported then the judgement of ‘unclear risk’ was made. Disagreements were solved initially via discussion between the two independent reviewers however a third reviewer (KD) was consulted for dispute resolution. ‘Risk of bias graphs’ were computed in Review Manager (RevMan) 5.3 (The Cochrane Collaboration) to include low, unclear and high risk for each domain.

### Statistical analysis

Outcome measures were quantified using mean difference between conditions with 95% confidence intervals (CI) which were used as the summary statistic. A random-effects meta-analysis was performed by AG, JM and KD using Comprehensive Meta-Analysis Software (version 3, Biostat, Englewood, NJ, USA). The inputted data included sample sizes, outcome measures with their respective SDs, and a correlation coefficient for within-participant measurements. These correlation coefficients were estimated from prior studies in our laboratory and were as follows: RER r = 0.78, absolute carbohydrate oxidation r = 0.70, absolute fat oxidation r = 0.81, relative carbohydrate oxidation r = 0.79, relative fat oxidation r = 0.79 [5, 6].

A negative mean difference indicates that hypoxic exposure was associated with a decrease in the respective outcome variable, while a positive mean difference indicates that hypoxic exposure was associated with an increase in the respective outcome variable. Heterogeneity between trials was assessed using the Chi-squared statistic, *I*-squared statistic and the Tau-squared statistic.

To examine whether any conclusions were dependent on a single study, sensitivity analyses was employed for each variable by repeating the analyses with each study omitted in turn.

Where the number of comparisons was suitable, meta-regression analysis was performed. This analysis was used to determine whether continuous or categorical data, including severity of hypoxic exposure, exercise intensity and pre-exercise nutritional state could explain the variation in the values observed between studies. Each moderator was analysed in a meta-regression independently to determine their relationship with the outcome variable. Where significant moderators were identified, multiple meta-regression analysis was conducted to determine if these remained significant, whilst controlling for all other moderators. All meta-regressions were performed using the restricted maximum likelihood (REML) method with Knapp-Hartung adjustment.

Duration of hypoxic exposure was categorised into acute and chronic as per each study’s description of their own exposure (acute < 44 h; chronic = 3–28 days). Overall mean differences, CIs and *p* values were reported for all variables during exercise matched to relative intensities. Meta-regression analysis was performed only on the outcome variable RER, as this represents the most appropriate measure to assess changes in the relative substrate contributions of carbohydrate and fat (i.e., physiological shifts in substrate utilisation). Meta-regression analysis of absolute fat and carbohydrate oxidation rates was deemed unnecessary based on these responses determining the RER values for each study.

### Exploration of small study effects

Small study effects were explored using funnel plots of mean difference versus standard errors [26], and by quantifying Egger’s linear regression intercept. A statistically significant Egger’s statistic (*p* < 0.05) indicates the presence of small study effects.

## Results

### Overview

**Fig. 1:**
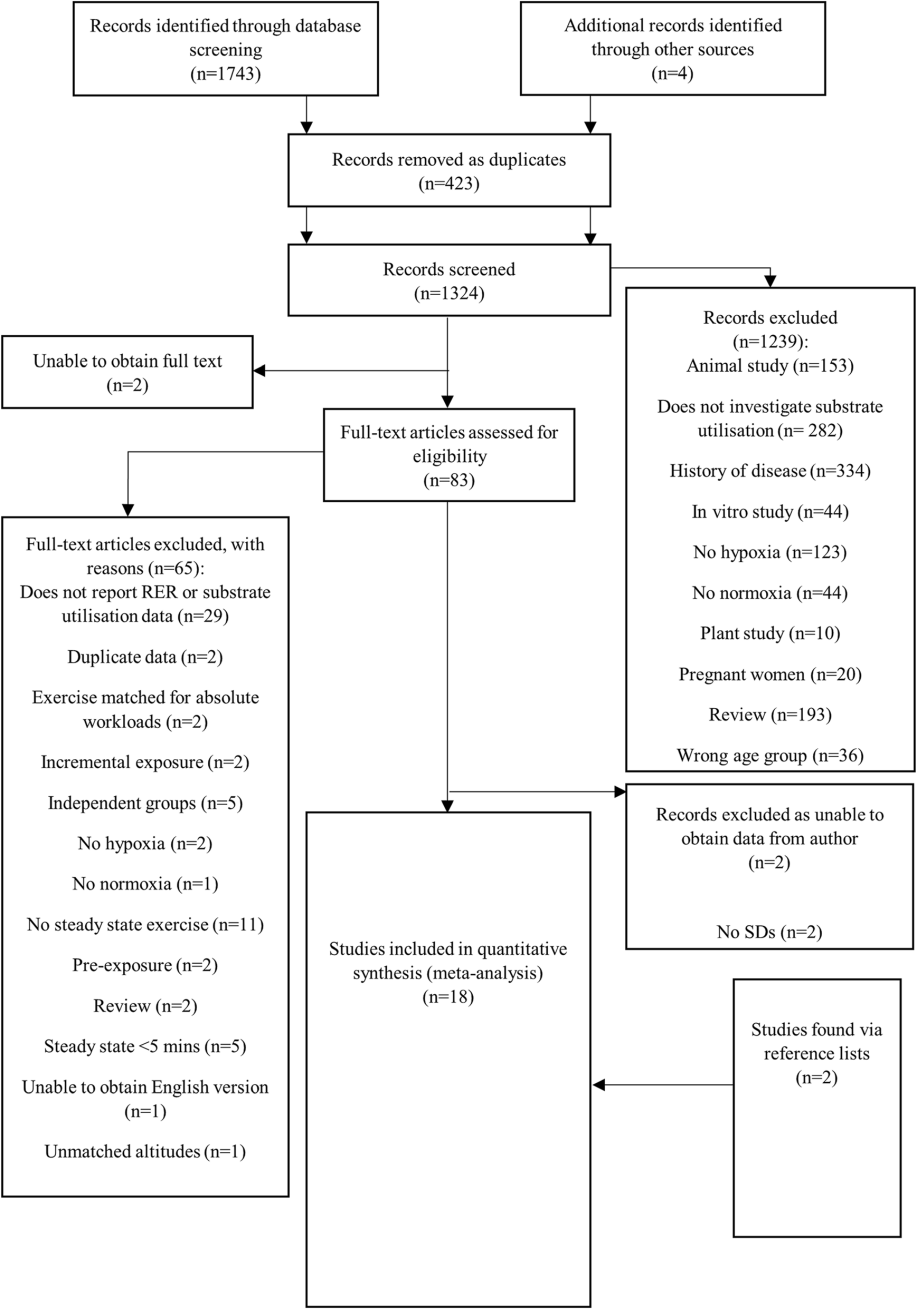
Flow chart of study selection

A total of 1743 studies published in peer reviewed scientific journals were initially identified through database screening and other sources. Following the full screening process, 18 studies were identified as suitable for the meta-analyses (Fig. 1). Within the 18 studies, a total of 58 comparisons between normoxic and hypoxic conditions were made for exercise matched for relative intensities. Of these 58 comparisons, 31 reported RER (Table 1) and 27 reported substrate utilisation (Table 2) ((absolute carbohydrate oxidatio*n* = 7, absolute fat oxidation = 6; relative carbohydrate oxidation = 7, relative fat oxidation = 7).

**Table 1:** Studies investigating RER during exercise matched for relative intensities in hypoxia, compared with normoxia

Study	Participants	Study design	Type of hypoxia	Altitude (m)	Duration of hypoxia	RER
Beidleman et al. (2002) [16]	8 (females)	Treadmill exercise at 70% relative VO_2max_ until volitional exhaustion	HH	4300	180 min	SL: 0.89 ± 0.04 AH: 0.85 ± 0.03
Beidleman et al. (2003) A [45]	6 (male = 5, female = 1)	15 min cycling at 40% relative VO_2max_	HH	4300	70 min	SL: 0.97 ± 0.02 AH: 0.88 ± 0.01
Beidleman et al. (2003) B [45]	6 (male = 5, female = 1)	15 min cycling at 70% relative VO_2max_	HH	4300	70 min	SL: 1.02 ± 0.02 AH: 1.00 ± 0.03
Bouissou et al. (1987) [46]	6 (males)	60 min cycling at 60% relative VO_2max_	HH	3000	80 min	SL: 0.99 ± 0.02 AH: 1.00 ± 0.01
Braun et al. (2000) [11]	15 (females)	30 min cycling at 65% relative VO_2max_	TA	4300	10 days	SL: 0.97 ± 0.01 CH: 0.94 ± 0.01
Friedmann et al. (2004) [47]	11 (males)	60 min running at ~ 82% relative VO_2max_	NH	2500	120 min	SL: 0.93 ± 0.02 AH: 0.98 ± 0.07
Fulco et al. (2005) A [48]	16 (males)	20 min cycling at 48% relative VO_2max_	TA	4300	3 days	SL: 0.91 ± 0.01 CH: 0.81 ± 0.01
Fulco et al. (2005) B [48]	16 (males)	20 min cycling at 48% relative VO_2max_	TA	4300	10 days	SL: 0.91 ± 0.01 CH: 0.80 ± 0.01
Fulco et al. (2005) C [48]	16 (males)	20 min cycling at 68% relative VO_2max_	TA	4300	3 days	SL: 0.93 ± 0.01 CH: 0.85 ± 0.01
Fulco et al. (2005) D [48]	16 (males)	20 min cycling at 68% relative VO_2max_	TA	4300	10 days	SL: 0.93 ± 0.01 CH: 0.84 ± 0.01
Hopkins et al. (2003) A [49]	6 (male = 1, female = 5)	5 min cycling at 30% relative VO_2max_	NH	3850	25 min	SL: 0.82 ± 0.02 AH: 0.86 ± 0.03
Hopkins et al. (2003) B [49]	6 (male = 1, female = 5)	5 min cycling at 60% relative VO_2max_	NH	3850	25 min	SL: 0.95 ± 0.02 AH: 0.96 ± 0.03
Katayama et al. (2010) [3]	7 (males)	30 min cycling at 50% relative VO_2max_	HH	2000	100 min	SL: 0.90 ± 0.01 AH: 0.91 ± 0.02
Lundby and Van Hall (2002) A [22]	8 (male = 6, female = 2)	60 min cycling at 50% relative VO_2max_	NH	4100	70 min	SL: 0.91 ± 0.01 AH: 0.92 ± 0.02
Lundby and Van Hall (2002) B [22]	8 (male = 6, female = 2)	60 min cycling at 50% relative VO_2max_	TA	4100	28 days	SL: 0.91 ± 0.01 CH: 0.91 ± 0.01
Maher et al. (1974) A [50]	8 (males)	10 min cycling at 75% relative VO_2max_	TA	4300	44 h	SL: 0.99 ± 0.01 AH: 0.98 ± 0.01
Maher et al. (1974) B [50]	8 (males)	10 min cycling at 75% relative VO_2max_	TA	4300	12 days	SL: 0.99 ± 0.01 CH: 0.98 ± 0.01
Matu et al. (2017) A [6]	12 (males)	60 min walking at 50% relative VO_2max_	NH	2150	5 h	SL: 0.88 ± 0.04 AH: 0.85 ± 0.06
Matu et al. (2017) B [6]	12 (males)	60 min walking at 50% relative VO_2max_	NH	4300	5 h	SL: 0.88 ± 0.04 AH: 0.85 ± 0.06
Messier et al. (2017) [51]	20 (males)	60 min cycling at ~ 67% relative VO_2max_	TA	2150	150 min	SL: 0.92 ± 0.06 AH: 0.97 ± 0.05
Noordhof et al. (2013) A [52]	16 (male)	6 min cycling at 45% relative VO_2max_	HH	1500	30 min	SL: 0.89 ± 0.03 AH: 0.92 ± 0.02
Noordhof et al. (2013) B [52]	16 (male)	6 min cycling at 55% relative VO_2max_	HH	1500	30 min	SL: 0.91 ± 0.04 AH: 0.94 ± 0.04
Noordhof et al. (2013) C [52]	16 (male)	6 min cycling at 65% relative VO_2max_	HH	1500	30 min	SL: 0.94 ± 0.04 AH: 0.98 ± 0.04
O’Hara et al. (2017) A [5]	7 (males)	5 min cycling at ~ 62% relative VO_2max_	HH	3375	155 min	SL: 0.92 ± 0.04 AH: 0.83 ± 0.04
O’Hara et al. (2017) B [5]	7 (males)	105 min cycling at ~ 74% relative VO_2max_	HH	3375	155 min	SL: 0.92 ± 0.03 AH: 0.84 ± 0.05
Peronnet et al. (2006) [4]	5 (males)	80 min cycling at 77% relative VO_2max_	HH	4300	110 min	SL: 0.93 ± 0.01 AH: 0.97 ± 0.01
Wyss et al. (1990) [53]	7 (males)	30 min running at ~ 79% relative VO_2max_	NH	3500	60 min	SL: 0.90 ± 0.04 AH: 0.93 ± 0.04
Young et al. (1987) A [54]	12 (males)	30 min cycling at 75% relative VO_2max_ (active between exercise tests)	TA	4300	< 24 h	SL: 0.84 ± 0.02 AH: 1.03 ± 0.01
Young et al. (1987) B [54]	12 (males)	30 min cycling at 75% relative VO_2max_ (active between exercise tests)	TA	4300	13 days	SL: 0.84 ± 0.02 CH: 1.03 ± 0.06
Young et al. (1987) C [54]	12 (males)	30 min cycling at 75% relative VO_2max_ (sedentary between exercise tests)	TA	4300	< 24 h	SL: 0.84 ± 0.01 AH: 1.05 ± 0.01
Young et al. (1987) D [54]	12 (males)	30 min cycling at 75% relative VO_2max_ (sedentary between exercise tests)	TA	4300	13 days	SL: 0.84 ± 0.01 CH: 1.13 ± 0.05

Values presented as mean ± SD. *HH* hypobaric hypoxia, *NH* normobaric hypoxia, *TA* terrestrial altitude, *SL* sea level, *AH* acute hypoxia, *CH* chronic hypoxia. A, B, C and D refer to the different trial arms of each study

**Table 2:** Studies investigating substrate utilisation during exercise matched for relative intensities in hypoxia compared with normoxia

Study	Participants	Study design	Type of hypoxia	Altitude (m)	Duration of exposure	Absolute substrate use (g.min^−1^)		Relative substrate use (%)	
						CHO oxidation	Fat oxidation	CHO oxidation	Fat oxidation
Braun et al. (2000) [11]	15 (females)	30 min cycling at 65% relative VO_2max_	TA	4300	10 days	SL:1.95 ± 0.11 CH:1.22 ± 0.09	NM	NM	NM
Lundby and Van Hall (2002) A [22]	8 (male = 6, female = 2)	60 min cycling at 50% relative VO_2max_	NH	4100	70 min	SL: 2.00 ± 0.20 AH: 1.70 ± 0.10	SL: 0.30 ± 0.01 AH: 0.20 ± 0.02	SL: 73.90 ± 2.00 AH: 75.50 ± 1.90	SL: 26.10 ± 2.00 AH: 24.50 ± 1.90
Lundby and Van Hall (2002) B [22]	8 (male = 6, female = 2)	60 min cycling at 50% relative VO_2max_	TA	4100	10 days	SL: 2.00 ± 0.20 CH: 1.70 ± 0.02	SL: 0.30 ± 0.01 CH: 0.30 ± 0.02	SL: 73.90 ± 2.00 CH: 74.40 ± 1.50	SL: 26.10 ± 2.00 CH: 25.60 ± 1.50
Matu et al. (2017) A [6]	12 (males)	60 min walking at 50% relative VO_2max_	NH	2150	5 h	SL: 1.56 ± 0.35 AH: 1.18 ± 0.34	SL: 0.41 ± 0.18 AH: 0.44 ± 0.21	SL: 62.80 ± 13.30 AH: 55.10 ± 18.90	SL: 37.20 ± 13.30 AH: 44.90 ± 18.90
Matu et al. (2017) B [6]	12 (males)	60 min walking at 50% relative VO_2max_	NH	4300	5 h	SL: 1.56 ± 0.35 AH: 0.87 ± 0.37	SL: 0.41 ± 0.18 AH: 0.38 ± 0.19	SL: 62.80 ± 13.30 AH: 50.80 ± 19.80	SL: 37.20 ± 13.30 AH: 49.20 ± 19.80
Morishima et al. (2014) A [55]	8 (males)	30 min cycling at 60% relative VO_2max_	NH	2700	7.5 h	NM	NM	SL: 60.00 ± 7.80 AH: 93.70 ± 2.50	SL: 40.00 ± 7.80 AH: 6.30 ± 2.50
O’Hara et al. (2017) B [5]	7 (males)	105 min cycling at ~ 74% relative VO_2max_	HH	3375	155 min	SL: 2.64 ± 0.50 AH: 1.47 ± 0.62	SL: 0.38 ± 0.19 AH: 0.63 ± 0.25	SL: 73.10 ± 13.10 AH: 48.80 ± 18.90	SL: 26.90 ± 13.10 AH: 51.20 ± 18.90
Peronnet et al. (2006) [4]	5 (males)	80 min cycling at 77% relative VO_2max_	HH	4300	110 min	SL:3.25 ± 0.13 AH:2.67 ± 0.10	SL:0.37 ± 0.05 AH: 0.10 ± 0.03	SL:78.10 ± 1.80 AH: 92.00 ± 2.10	SL: 21.90 ± 1.80 AH: 8.00 ± 2.10

Values presented as mean ± SD. *HH* hypobaric hypoxia, *NH* normobaric hypoxia, *TA* terrestrial altitude, *SL* sea level, *AH* acute hypoxia, *CH* chronic hypoxia, *CHO* carbohydrate, *NM* not measured. A and B refer to the different trial arms of each study

### Participant demographics and study characteristics

Of the 170 participants included in the analysis, 146 were male (86%) and 24 were female (14%). Age was reported in all studies and ranged from 20 to 39 years (mean = 27 years). BMI was reported in 15 of the 18 studies and ranged from 21.3 to 28.6 kg·m^− 2^ (mean = 23.4 kg·m^− 2^). VO_2max_ was reported in 17 of the 18 studies and ranged between 2.61 and 4.99 L·min^− 1^ (mean = 3.75 L·min^− 1^).

Exercise duration ranged from 5 to 105 min (mean = 39 min). Participants in normoxic trials performed exercise at intensities ranging from 30 to 82% of normoxic VO_2max_ (mean = 61% SL VO_2max_) and hypoxic trials were performed at 30–83% of hypoxic specific VO_2max_ (mean = 61% hypoxic VO_2max_). The severity of hypoxia quantified in meters, ranged from 1500 m to 4300 m (mean = 3499 m). Feeding status was only specified in 26 out of 31 comparisons (fasted = 8; fed = 18).

### Meta-analysis

Individual study statistics and results for each variable are provided in the supplementary tables (Additional files 2, 3, 4, 5 and 6).

### RER

**Fig. 2:**
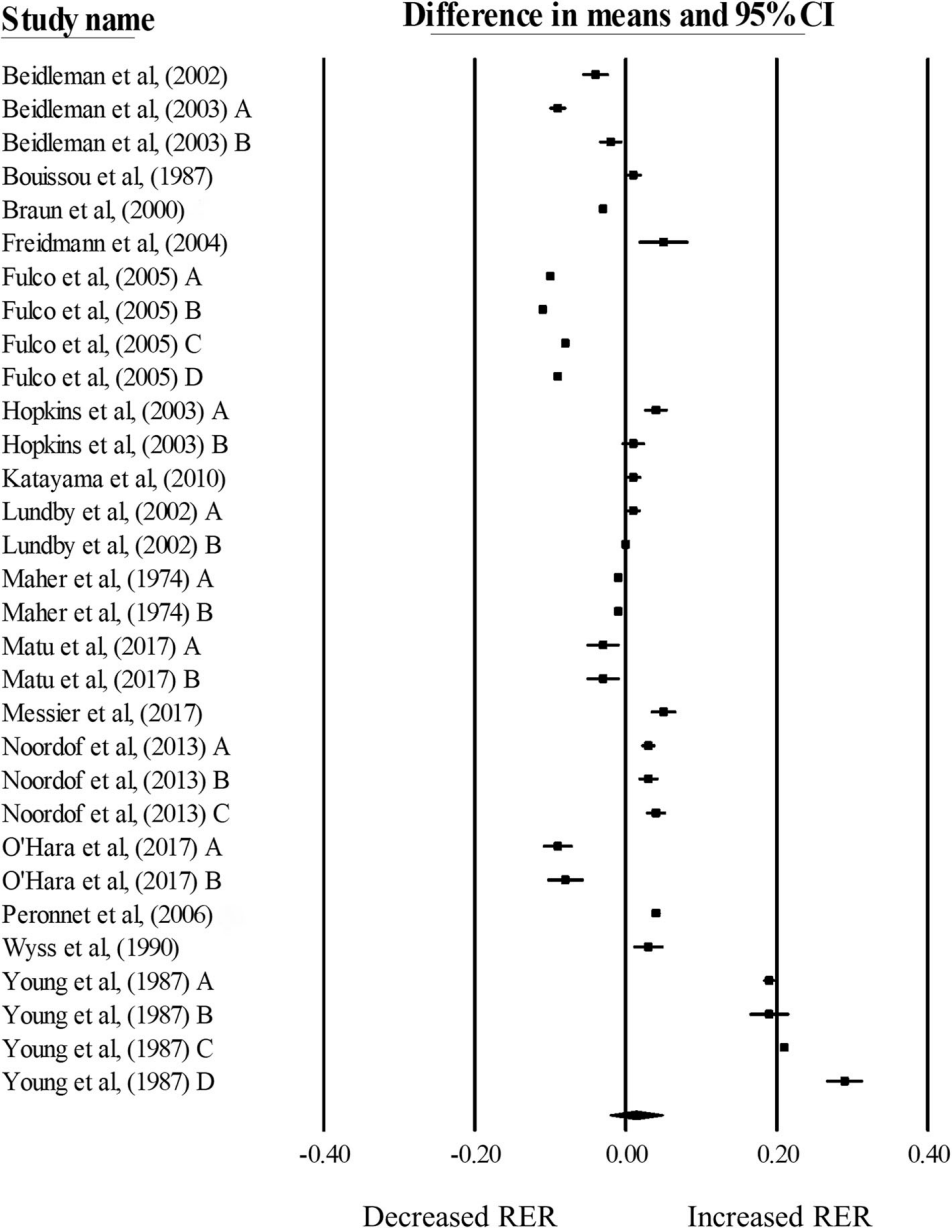
Forest plot of mean differences (means ± 95% CI) for studies investigating the effects of hypoxia on RER during exercise matched for relative intensities. The size of the circle represents the relative weight of the trial. CIs are represented by a horizontal line through their representative circles. The diamond quantifies the overall mean difference (means ± 95% CI). A, B, C and D refer to the different trial arms of each study. Details of which are provided in Table 1

There was no significant change in RER during exercise matched for relative intensities in hypoxia, compared with normoxia (mean difference: 0.01, 95% CI: -0.02 to 0.05; *n* = 31, *p* = 0.45; Fig. 2). The degree of heterogeneity was found to be high between studies (*I*^2^ = 99.87%, Q = 27,768, τ^2^ = 0.01, d_f_ = 30). Sensitivity analysis revealed minor changes only, and these changes did not substantially alter the overall mean difference. Inspection of the funnel plot and Egger’s regression intercept revealed that there was little evidence of small study effects (intercept = 12.61, 95% CI: -5.87 to 31.08; *p* = 0.17).

Meta-regression analysis (Table 3) indicated a difference in RER responses between participants in the fasted and fed state (*p* < 0.01), with participants in the fasted state demonstrating a decreased RER, and those in the fed state demonstrating an increased RER during exercise matched for relative exercise intensity in hypoxia, compared with normoxia. Using exercise intensity as a moderator, a greater exercise intensity was associated with a greater increase in RER during exercise matched to relative intensity in hypoxia, compared with normoxia. The slope of the regression was significantly positive (*p* = 0.04), with a standardised increase of 0.0033 units, for every percentage increase in exercise intensity. When the significant variables from the bivariate analysis were entered into the multiple regression models, both pre-exercise nutritional state and exercise intensity remained significant. The multiple regression model explained 42% of the variance observed (R^2^ = 0.42).

**Table 3:** Summary of moderator variables from the single and multiple meta-regression model for RER in response to hypoxic exposure during exercise matched for relative intensities

Moderator variable RER (relative) (*n* = 31)	*p* value	Comparison	Multiple regression *p* value
Pre-existing nutritional state	< 0.01	Fasted (*n* = 8, MD − 0.07, 95% CI − 0.09 to − 0.06) Fed (*n* = 18, MD 0.06, 95% CI 0.01 to 0.10)	< 0.01
Carbohydrate supplementation during exercise	0.22	Yes (*n* = 4, MD − 0.04, 95% CI − 0.09 to 0.01) No (*n* = 27, MD 0.02, 95% -0.02 to 0.06)	N/A
Exercise mode	0.60	Cycling (*n* = 26, MD 0.02, 95% CI − 0.02 to 0.05) Running (*n* = 5, MD − 0.01, 95% CI − 0.04 to 0.03)	N/A
Duration of hypoxic exposure	0.67	Acute (*n* = 22, MD 0.02, 95% CI − 0.03 to 0.06) Chronic (*n* = 9, MD 0.00, 95% CI − 0.03 to 0.04)	N/A
Type of hypoxia	0.96	Simulated normobaric hypoxia (n = 7, MD 0.01, 95% CI − 0.01 to 0.03) Simulated hypobaric hypoxia (n = 9, MD 0.00, 95% CI − 0.03 to 0.03) Terrestrial altitude (*n* = 15, MD 0.02, 95% CI − 0.03 to 0.07)	N/A
Percentage male	0.43	Meta-regression percentage male vs. MD (slope 0.0006, 95% CI − 0.0009 to 0.0021)	N/A
Exercise intensity	0.04	Meta-regression of exercise intensity vs. MD (slope 0.0033, 95% CI 0.0002 to 0.0065)	0.049
Exercise duration	0.78	Meta-regression of exercise duration vs. MD (slope − 0.0002, 95% CI − 0.0018 to 0.014)	N/A
Altitude height	0.90	Meta-regression of altitude height vs. ES (slope − 0.00, 95% CI − 0.00 to 0.00)	N/A

### Relative carbohydrate and fat oxidation rates

There was no significant change in relative carbohydrate oxidation rates during exercise matched for relative intensities in hypoxia, compared with normoxia (mean difference: 1.74, 95% CI: -4.76 to 8.25%; *n* = 7, *p* = 0.60; Additional file 7). The degree of heterogeneity was found to be high between studies (*I*^2^ = 99.09%, Q = 659, τ^2^ = 71.00, d_f_ = 6). Sensitivity analysis revealed minor changes only, and these changes did not substantially alter the overall mean difference. Inspection of the funnel plot and Egger’s regression intercept revealed that there was little evidence of small study effects (intercept = 0.69, 95% CI: -16.79 to 18.17; *p* = 0.92).

There was no significant change in relative fat oxidation during exercise matched for relative intensities in hypoxia, compared with normoxia (mean difference: -1.74, 95% CI = − 8.25 to 4.76%, *n* = 7, *p* = 0.60; Additional file 8). The degree of heterogeneity was found to be high between studies (*I*^2^ = 99.09%, Q = 659, τ^2^ = 71.00, d_f_ = 6). Sensitivity analysis revealed minor changes only, and these changes did not substantially alter the overall mean difference. Inspection of the funnel plot and Egger’s regression intercept revealed that there was little evidence of small study effects (intercept = − 0.69, 95% CI: -18.17 to 16.79; *p* = 0.92).

### Absolute carbohydrate and fat oxidation rates

**Fig. 3:**
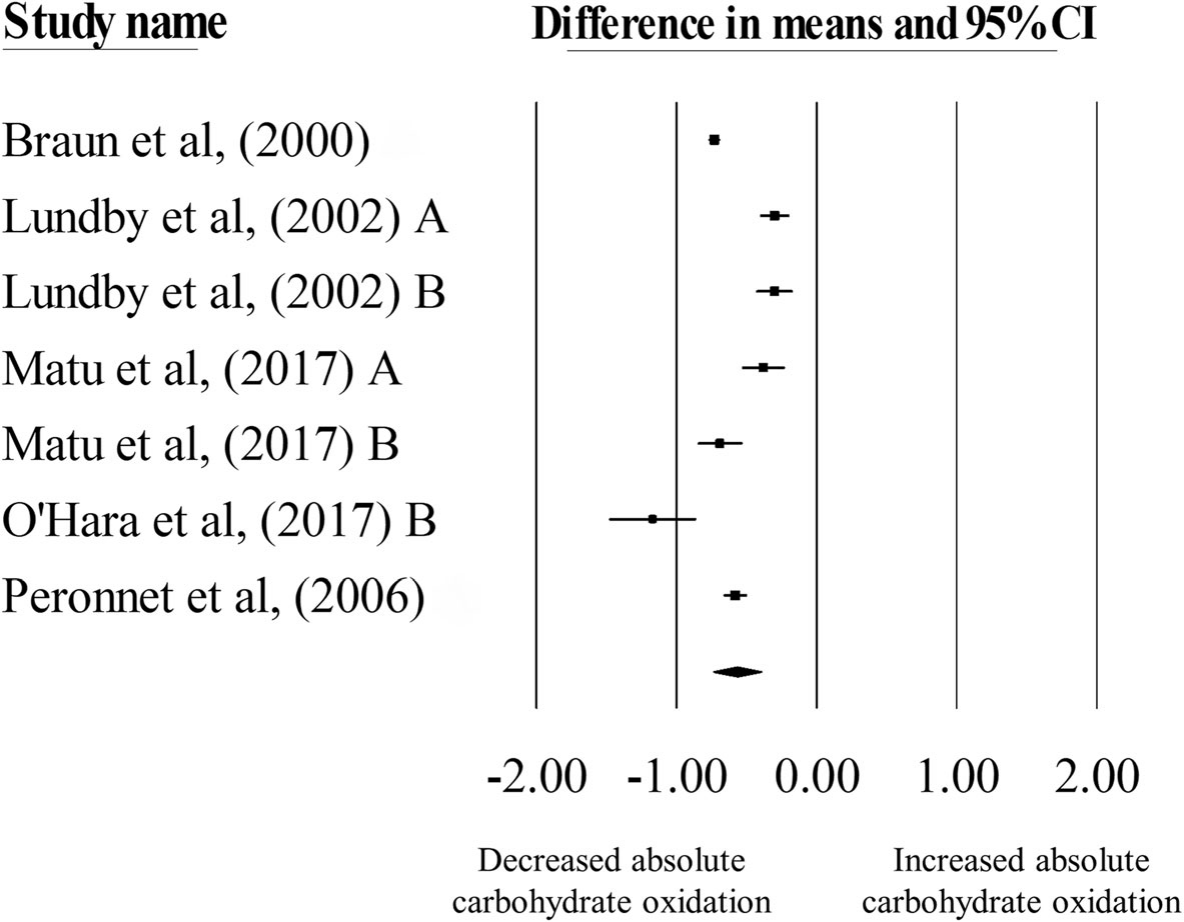
Forest plot of mean differences (means ± 95% CI) for studies investigating the effects of hypoxia on absolute carbohydrate oxidation during exercise matched for relative intensities. The size of the circle represents the relative weight of the trial. CIs are represented by a horizontal line through their representative circles. The diamond quantifies the overall mean difference (means ± 95% CI). A and B refer to the different trial arms of each study. Details of which are provided in Table 2

There was a significant decrease in absolute carbohydrate oxidation rates during exercise matched for relative intensities in hypoxia, compared with normoxia (mean difference: − 0.57 g·min^− 1^, 95% CI: -0.74 to − 0.40 g·min^− 1^; *n* = 7; *p* < 0.01; Fig. 3). The degree of heterogeneity was found to be high between studies (*I*^2^ = 94.66%, Q = 112, τ^2^ = 0.05, d_f_ = 6). Sensitivity analysis revealed minor changes only, and these changes did not substantially alter the overall mean difference. Inspection of the funnel plot and Egger’s regression intercept revealed little evidence of small study effects (intercept = 3.25, 95% CI: -4.34 to 10.84; *p* = 0.32).

**Fig. 4:**
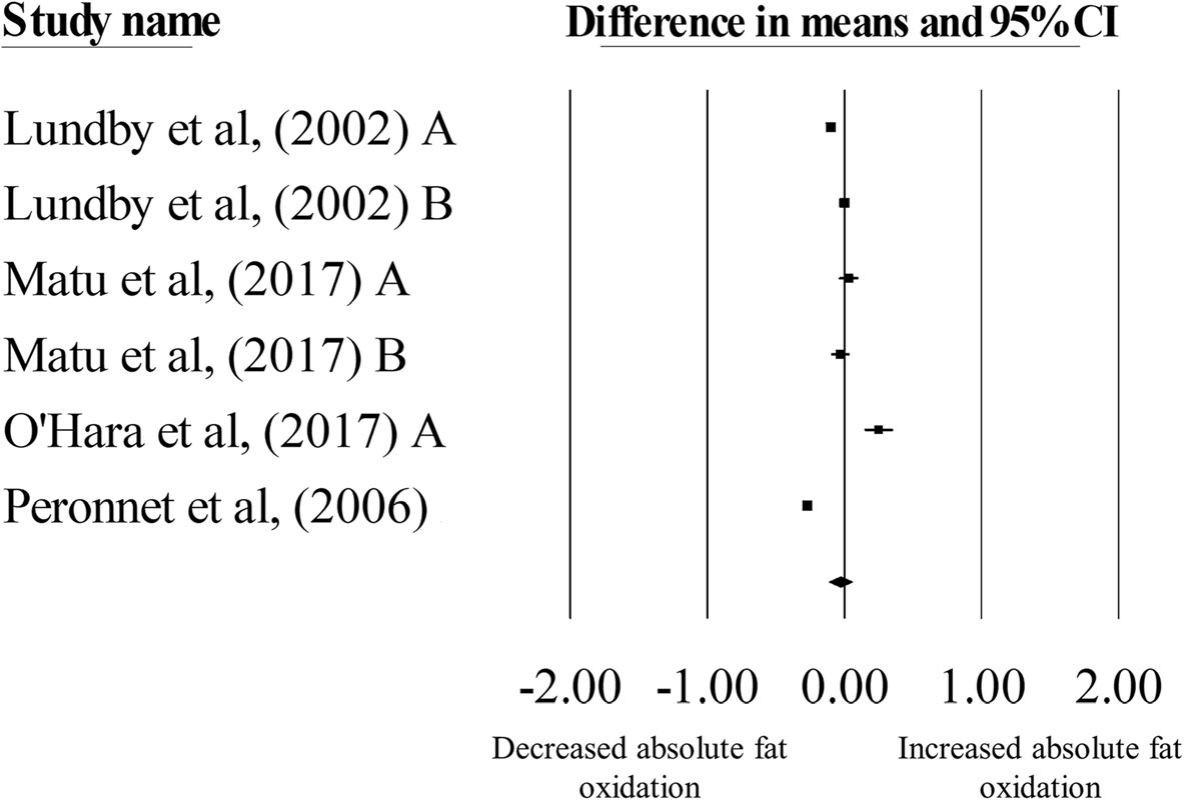
Forest plot of mean differences (means ± 95% CI) for studies investigating the effects of hypoxia on absolute fat oxidation during exercise matched for relative intensities. The size of the circle represents the relative weight of the trial. CIs are represented by a horizontal line through their representative circles. The diamond quantifies the overall mean difference (means ± 95% CI). A and B refer to the different trial arms of each study. Details of which are provided in Table 2

There was no significant change in absolute fat oxidation during exercise matched for relative intensities in hypoxia, compared with normoxia (mean difference: − 0.03 g·min^− 1^, 95% CI: -0.11 to 0.05 g·min^− 1^; *n* = 6, *p* = 0.44; Fig. 4). The degree of heterogeneity was found to be high between studies (*I*^2^ = 99.01%, Q = 506, τ^2^ = 0.01, d_f_ = 5). Sensitivity analysis revealed minor changes only, and these changes did not substantially alter the overall mean difference. Inspection of the funnel plot and Egger’s regression intercept revealed that there was some evidence of small study effects (intercept = − 5.96, 95% CI: -13.14 to 1.25; *p* = 0.08).

### Risk of bias

**Fig. 5:**
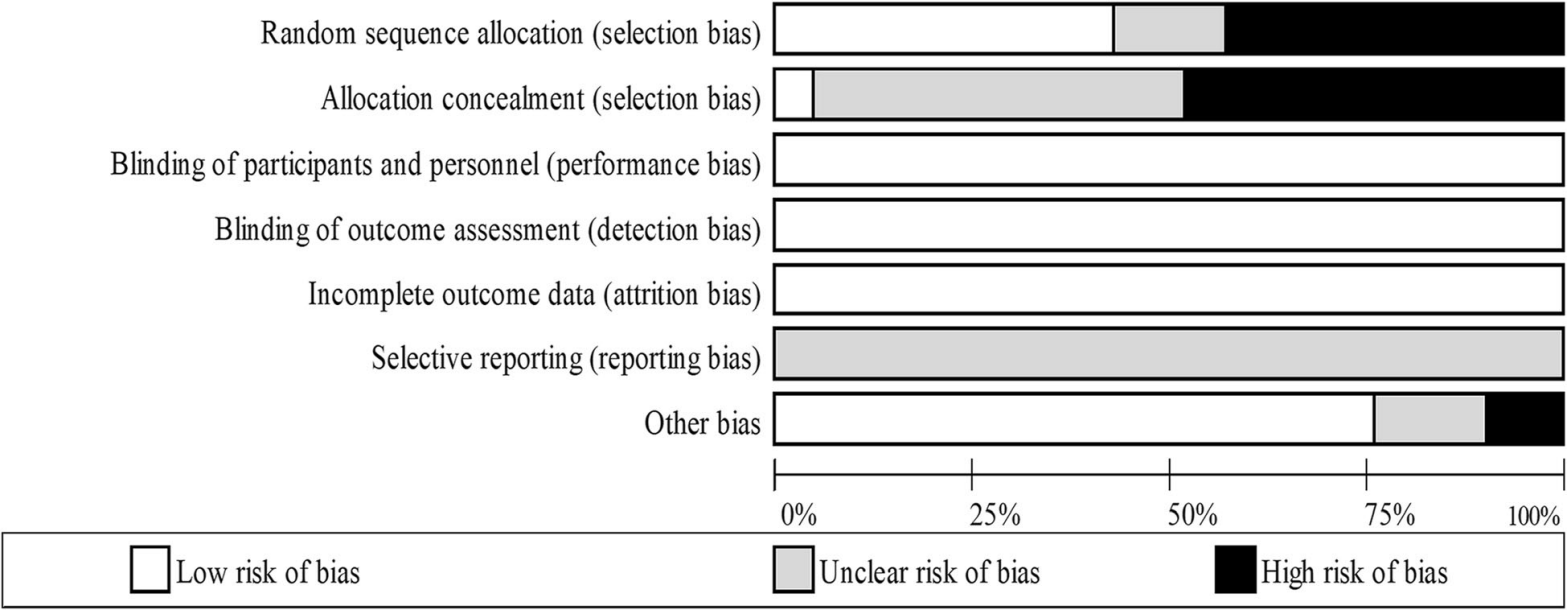
Assessment of risk of bias (Cochrane’s collaboration tool)

Since many of the studies were high altitude expeditions, certain biases were often unavoidable such as blinding of participants and personnel (Fig. 5). However, it was deemed that some of these biases could not affect the outcome variable and were therefore classified as low risk. In addition, all included studies were not clinically registered, therefore it is not possible to determine if all outcome variables were reported, therefore selective reporting bias was listed as unclear.

## Discussion

The purpose of this systematic review and meta-analysis was to examine the effects of hypoxic exposure on substrate oxidation during exercise matched to relative intensities. There was no consistent change in relative carbohydrate or fat contribution to energy provision during exercise matched for relative intensities in hypoxia, compared with normoxia. These findings are particularly pertinent as, in contrast to exercise matched to absolute intensities, exercise matched to relative intensities isolates the effect of hypoxia by normalising the exercise intensities between conditions [22]. Additionally, the heterogeneous findings of the current literature may be explained by a number of differing experimental characteristics, such as pre-exercise nutritional status and exercise intensity.

There was no significant change observed in RER during exercise matched for relative intensities in hypoxia, compared with normoxia. In addition, there was no significant change in relative carbohydrate or fat oxidation in the same circumstances. It was deemed that RER was the most useful outcome variable due the largest number of comparisons as a result of being the most frequently reported in the literature. As expected, reduced absolute carbohydrate rates were observed in hypoxia during exercise matched to relative intensities, due to the lower absolute workload [27] performed in hypoxia than normoxia and the subsequent reduction in energy expenditure. However, no significant change in absolute fat oxidation was observed in hypoxia, likely due to the limited changes in the contribution from this fuel source at moderate (40–55% VO_2max_) exercise intensities [28].

The current review found that an increase in RER was induced during exercise matched for relative intensities in hypoxia compared with normoxia when participants were in the fed state. Alternatively, a decrease in RER was induced when in the fasted state. The increase in endogenous carbohydrate stores as a result of feeding may facilitate a hypoxic-induced physiological demand for increased carbohydrate oxidation, thus potentiating the fuel shift. The mobilisation and oxidation of these stores may be augmented by the synergistic effect of feeding [29] and hypoxia [3] on sympathetic nervous system activity (i.e. increased secretion of epinephrine and norepinephrine) and resultant increases in gluconeogenesis and glycogenolysis. Interestingly, recent evidence also suggests that the rise in circulating insulin concentrations after feeding may increase carbohydrate oxidation from muscle glycogen stores, even before the ingested carbohydrate has been transported into the muscle [30]. This is supported by previous work demonstrating a reduction in muscle glycogen concentrations one-hour after consuming a mixed macronutrient meal, before increasing again in the subsequent hours [31]. This increase in insulin concentrations after a pre-exercise meal may be potentiated by hypoxia [6], thereby enhancing the inhibition of lipolysis and FFA mobilisation [32] to increase carbohydrate oxidation. In contrast, fasted exercise may elicit a decrease in RER via the enhanced activation of PPARα due to both hypoxia [8] and fasting [33]. The synergistic effect of both factors may further disrupt glycolysis [9] and enable greater fat flux [10].

In addition, an increased RER was observed during exercise matched to relative intensities in hypoxia, compared with normoxia, during exercise performed at higher intensities. This effect may be mediated by the hypoxic effect of altitude and high intensity exercise, augmenting skeletal muscle hypoxia [34]. The mechanisms associated with these changes are likely explained as per the physiological response to increased exercise intensities in normoxic environments. In this regard, higher exercise intensities induce a reduction in adipose tissue blood flow, which may attenuate the release of FFA resulting in decreased delivery to the contracting muscle [35]. Further, greater exercise intensities stimulate greater flux through the glycolytic pathway and pyruvate dehydrogenase complex (PDC) than flux through the tricarboxylic acid cycle, resulting in the accumulation of acetyl coA [36]. The subsequent acylation of the carnitine pool has been suggested to result in a marked decrease in muscle free carnitine and downregulation of carnitine palmitoyltransferase I (CPT-1), the enzyme responsible for transporting long chain fatty acids into the mitochondrial matrix [28]. Alternatively, the effect of hypoxia on the sympathetic nervous system may be potentiated by greater exercise intensities, enabling greater carbohydrate oxidation due to increased glycogenolysis, a result of enhanced glycogen phosphorylase activity, sarcoplasmic Ca^2+^, inorganic phospohate and cyclic AMP [37, 38]. Numerous mechanisms are proposed to explain the reduction in FFA oxidation with increasing exercise intensities [39], however detailed discussion of all theories is out of the scope of this review.

The large between study heterogeneity in relation to RER during exercise matched to relative intensities was explained in part by pre-exercise nutritional state and exercise intensity (~ 42%). The remaining, unexplained heterogeneity may highlight some limitations of the present meta-analysis. Results from a meta-regression are indicative of a between-study relationship, however due to confounding bias (i.e. one experimental characteristic may reflect a true association with other correlated, known or unknown characteristics), this relationship may not be replicated within-studies. This is termed aggregation bias. As such, moderator analysis should be regarded as hypothesis gathering, rather than hypothesis testing [40]. These moderators should therefore subsequently be investigated using a within-measures design via randomised controlled trials, generating causal, rather than observational relationships. Further, the unexplained heterogeneity may be due to methodological heterogeneity (i.e. study quality/measurement error) or insufficient trials to generate the appropriate power to fully explain the heterogeneity. A greater quantity and quality of research regarding substrate oxidation during hypoxia would help to further explain the heterogeneity between trials Further research is required to confirm the findings from this meta-analysis and quantify the influence of the fasted and fed state and exercise intensity on substrate utilisation in hypoxia.

The present meta-analysis provides clarity, and therefore facilitates an accurate interpretation, of the current literature. These findings may inform nutritional strategies for mountaineers, military personnel and athletes during exposure to altitude, subsequently limiting the detrimental exercise performance experienced in such conditions. The performance benefits of maintaining exogenous carbohydrate oxidation and/or endogenous carbohydrate stores via pre-exercise carbohydrate consumption in normoxia are well documented [41]. As such, findings from this review suggest that a physiological drive for carbohydrate oxidation in hypoxia may be facilitated by an increased carbohydrate intake prior to exercise, in order to avoid an accelerated depletion of muscle glycogen, and shift back to the less efficient oxidation of fat [35]. In contrast, the use of low carbohydrate intake strategies to enhance endurance training metabolic adaptations is growing in popularity [42] and findings from the present meta-analysis may have implications for such strategies. Specifically, the combined effect of training in hypoxia in a glycogen depleted state may potentiate the metabolic adaptations of ‘training low’. Alternatively, a number of studies have demonstrated that changes in substrate utilisation during exercise in hypoxia may have implications for metabolic disease programmes [43, 44]. As such, the clinical translation of the current study warrants further investigation.

Despite the important findings observed in the current meta-analysis, some notable limitations must be acknowledged. First, the equivocal findings observed in RER and relative substrate oxidation reflects the heterogeneity in the literature, rather than an absence of change in substrate oxidation due to hypoxic exposure. This heterogeneity is likely due to differing experimental characteristics between studies and although moderator analysis was employed to identify these factors, these findings should be interpreted with caution. Second, the physiological determinants of substrate oxidation (e.g. hormonal factors), were not quantified and therefore physiological mechanisms were difficult to elucidate. This was, however, beyond the scope of this study. Finally, despite an extensive search returning 1743 records, we cannot guarantee that our search was completely exhaustive of the relevant literature. However, should the primary or secondary aim of a study be related to this area, they are likely to have been detected in our search.

## Conclusions

This meta-analysis did not demonstrate a consistent change in relative carbohydrate or fat contribution to energy provision during exercise matched for relative intensities in hypoxia, compared with normoxia. These findings reflect the heterogeneity in the current literature. A metabolically efficient shift to carbohydrate oxidation may be induced by consumption of a pre-exercise meal and a higher exercise intensity. A significant amount of between-study heterogeneity could not be explained by the moderators used in this meta-analysis, highlighting the need for future research to further investigate moderators of this effect in a randomised and controlled fashion.

## Additional files

Additional file 1: Search strategy and key words. (DOCX 13 kb)

Additional file 2: Individual study statistics for studies evaluating RER during exercise matched for relative intensities in hypoxia compared with normoxia. A, B, C and D refer to the different trial arms of each study. Details of which are provided in Table 1. (DOCX 26 kb)

Additional file 3: Individual study statistics for studies evaluating relative carbohydrate oxidation during exercise matched for relative intensities in hypoxia compared with normoxia. A and B refer to the different trial arms of each study. Details of which are provided in Table 2. (DOCX 15 kb)

Additional file 4: Individual study statistics for studies evaluating relative fat oxidation during exercise matched for relative intensities in hypoxia compared with normoxia. A and B refer to the different trial arms of each study. Details of which are provided in Table 2. (DOCX 15 kb)

Additional file 5: Individual study statistics for studies evaluating absolute carbohydrate oxidation during exercise matched for relative intensities in hypoxia compared with normoxia. A and B refer to the different trial arms of each study. Details of which are provided in Table 2. (DOCX 15 kb)

Additional file 6: Individual study statistics for studies evaluating absolute fat oxidation during exercise matched for relative intensities in hypoxia compared with normoxia. A and B refer to the different trial arms of each study. Details of which are provided in Table 2. (DOCX 15 kb)

Additional file 7: Forest plot of mean differences (means ± 95% CI) for studies investigating relative carbohydrate oxidation during exercise matched for relative intensities in hypoxia compared with normoxia. The size of the circle represents the relative weight of the trial. CIs are represented by a horizontal line through their representative circles. The diamond quantifies the overall mean difference (means ± 95% CI). A and B refer to the different trial arms of each study. Details of which are provided in Table 2. (TIF 381 kb)

Additional file 8: Forest plot of mean differences (means ± 95% CI) for studies investigating relative fat oxidation during exercise matched for relative intensities in hypoxia compared with normoxia. The size of the circle represents the relative weight of the trial. CIs are represented by a horizontal line through their representative circles. The diamond quantifies the overall mean difference (means ± 95% CI). A and B refer to the different trial arms of each study. Details of which are provided in Table 2. (TIF 436 kb)

## Abbreviations

AH: Acute hypoxia
CH: Chronic hypoxia
CHO: Carbohydrate
CI: Confidence interval
CPT-1: Carnitine palmitoyltransferase
D1: mean of group 1 – mean of total group
D2: mean of group 2 – mean of total group
FFA: Free fatty acid
HH: Hypobaric hypoxia
HIF-1 α: Hypoxic-inducible factor 1 alpha
MD: Mean difference
n^1^: Sample size of group 1
n^2^: Sample size of group 2
NH: Normobaric hypoxia
NM: Not measured
PPARα: Peroxisome proliferator-activated receptor alpha
PRISMA: Preferred Reporting Items for Systematic Review and Meta-analyses
RER: Respiratory exchange ratio
S1: Standard deviation of group 1
S2: Standard deviation of group 2
SD: Standard deviation
SL: Sea level
TA: Terrestrial altitude
