# Supplementary material for: The effects of environmental hypoxia on substrate utilisation during exercise: a meta-analysis
Source: J Int Soc Sports Nutr. 2019 Feb 27;16:10. doi: 10.1186/s12970-019-0277-8 (PMC6391781; doi:10.1186/s12970-019-0277-8)
Supplement: Supplementary file 1 — Search strategy and key words. (DOCX 13 kb) [file 12970_2019_277_MOESM1_ESM.docx]

Search terms:

1. Exercise
2. Time trial
3. Time to exhaustion
4. Walking
5. Cycling
6. Trekking
7. Swimming
8. Skiing
9. Running

AND

1. Hypoxia
2. Altitude
3. Hypoxemia
4. Hypoxic

AND

1. Substrate
2. Carbohydrate
3. CHO
4. Fat
5. Glucose
6. Glycogen
7. Lipid

AND

1. Oxidation
2. Utilisation
3. Metabolism
4. Respiratory exchange ratio
5. RER

Searches

1. 1 + (10 or 11 or 12 or 13) + (14 or 15 or 15 or 16 or 17 or 18 or 19 or 20) + (21 or 22 or 23 or 24 or 25)
2. 2 + (10 or 11 or 12 or 13) + (14 or 15 or 15 or 16 or 17 or 18 or 19 or 20) + (21 or 22 or 23 or 24 or 25)
3. 3 + (10 or 11 or 12 or 13) + (14 or 15 or 15 or 16 or 17 or 18 or 19 or 20) + (21 or 22 or 23 or 24 or 25)
4. 4 + (10 or 11 or 12 or 13) + (14 or 15 or 15 or 16 or 17 or 18 or 19 or 20) + (21 or 22 or 23 or 24 or 25)
5. 5 + (10 or 11 or 12 or 13) + (14 or 15 or 15 or 16 or 17 or 18 or 19 or 20) + (21 or 22 or 23 or 24 or 25)
6. 6 + (10 or 11 or 12 or 13) + (14 or 15 or 15 or 16 or 17 or 18 or 19 or 20) + (21 or 22 or 23 or 24 or 25)
7. 7 + (10 or 11 or 12 or 13) + (14 or 15 or 15 or 16 or 17 or 18 or 19 or 20) + (21 or 22 or 23 or 24 or 25)
8. 8 + (10 or 11 or 12 or 13) + (14 or 15 or 15 or 16 or 17 or 18 or 19 or 20) + (21 or 22 or 23 or 24 or 25)
9. 9 + (10 or 11 or 12 or 13) + (14 or 15 or 15 or 16 or 17 or 18 or 19 or 20) + (21 or 22 or 23 or 24 or 25)
