# Supplementary material for: The effects of environmental hypoxia on substrate utilisation during exercise: a meta-analysis
Source: J Int Soc Sports Nutr. 2019 Feb 27;16:10. doi: 10.1186/s12970-019-0277-8 (PMC6391781; doi:10.1186/s12970-019-0277-8)
Supplement: Supplementary file 2 — Individual study statistics for studies evaluating RER during exercise matched for relative intensities in hypoxia compared with normoxia. A, B, C and D refer to the different trial arms of each study. Details of which are provided in Table 1. (DOCX 26 kb) [file 12970_2019_277_MOESM2_ESM.docx]

**Additional file 2. Individual study statistics for studies evaluating RER during exercise matched for relative intensity in hypoxia compared with normoxia. A, B, C and D refer to the different trial arms of each study. Details of which are provided in table 1.**

| Study | Mean difference | Standard error | Variance | Lower 95% confidence interval | Upper 95% confidence interval | p-value | Z | Weight |
| --- | --- | --- | --- | --- | --- | --- | --- | --- |
| Beidleman et al, (2002) | -0.04 | 0.01 | 0.00 | -0.06 | -0.02 | <0.01 | -4.51 | 3.22 |
| Beidleman et al, (2003) A | -0.09 | 0.01 | 0.00 | -0.10 | -0.08 | <0.01 | -16.08 | 3.23 |
| Beidleman et al, (2003) B | -0.02 | 0.01 | 0.00 | -0.04 | 0.00 | 0.01 | -2.57 | 3.23 |
| Bouissou et al, (1987) | 0.01 | 0.01 | 0.00 | 0.00 | 0.02 | 0.07 | 1.79 | 3.23 |
| Braun et al, (2000) | -0.03 | 0.00 | 0.00 | -0.03 | -0.03 | <0.01 | -17.52 | 3.24 |
| Freidmann et al, (2004) | 0.05 | 0.02 | 0.00 | 0.02 | 0.08 | <0.01 | 2.97 | 3.15 |
| Fulco et al, (2005) | -0.10 | 0.00 | 0.00 | -0.10 | -0.10 | <0.01 | -60.30 | 3.24 |
| Fulco et al, (2005) | -0.11 | 0.00 | 0.00 | -0.11 | -0.11 | <0.01 | -66.33 | 3.24 |
| Fulco et al, (2005) | -0.08 | 0.00 | 0.00 | -0.08 | -0.08 | <0.01 | -48.24 | 3.24 |
| Fulco et al, (2005) | -0.09 | 0.00 | 0.00 | -0.09 | -0.09 | <0.01 | -54.27 | 3.24 |
| Hopkins et al, (2003) | 0.04 | 0.01 | 0.00 | 0.02 | 0.06 | <0.01 | 5.14 | 3.23 |
| Hopkins et al, (2003) | 0.01 | 0.01 | 0.00 | -0.01 | 0.03 | 0.20 | 1.28 | 3.23 |
| Katayama et al, (2010) | 0.01 | 0.01 | 0.00 | 0.00 | 0.02 | 0.05 | 1.93 | 3.24 |
| Lundby et al, (2002) A | 0.01 | 0.00 | 0.00 | 0.00 | 0.02 | 0.04 | 2.06 | 3.24 |
| Lundby et al, (2002) B | 0.00 | 0.00 | 0.00 | 0.00 | 0.00 | 1.00 | 0.00 | 3.24 |
| Maher et al, (1974) A | -0.01 | 0.00 | 0.00 | -0.01 | -0.01 | <0.01 | -4.26 | 3.24 |
| Maher et al, (1974) B | -0.01 | 0.00 | 0.00 | -0.01 | -0.01 | <0.01 | -4.26 | 3.24 |
| Matu et al, (2017) A | -0.03 | 0.01 | 0.00 | -0.05 | -0.01 | 0.01 | -2.72 | 3.20 |
| Matu et al, (2017) B | -0.03 | 0.01 | 0.00 | -0.05 | -0.01 | 0.01 | -2.72 | 3.20 |
| Messier et al, (2017) | 0.05 | 0.01 | 0.00 | 0.03 | 0.07 | <0.01 | 5.93 | 3.22 |
| Noordhof et al, (2013) A | 0.03 | 0.00 | 0.00 | 0.02 | 0.04 | <0.01 | 6.29 | 3.24 |
| Noordhof et al, (2013) B | 0.03 | 0.01 | 0.00 | 0.02 | 0.04 | <0.01 | 4.52 | 3.23 |
| Noordhof et al, (2013) C | 0.04 | 0.01 | 0.00 | 0.03 | 0.05 | <0.01 | 6.03 | 3.23 |
| O’Hara et al, (2017) A | -0.09 | 0.01 | 0.00 | -0.11 | -0.07 | <0.01 | -8.97 | 3.21 |
| O’Hara et al, (2017) B | -0.08 | 0.01 | 0.00 | -0.10 | -0.06 | <0.01 | -6.50 | 3.19 |

**Additional file 2. Continued…**

| Study | Mean difference | Standard error | Variance | Lower 95% confidence interval | Upper 95% confidence interval | p-value | Z | Weight |
| --- | --- | --- | --- | --- | --- | --- | --- | --- |
| Peronnet et al, (2006) | 0.04 | 0.00 | 0.00 | 0.03 | 0.05 | <0.01 | 13.48 | 3.24 |
| Wyss et al, (1990) | 0.03 | 0.01 | 0.00 | 0.01 | 0.05 | <0.01 | 2.99 | 3.21 |
| Young et al, (1987) A | 0.19 | 0.00 | 0.00 | 0.18 | 0.20 | <0.01 | 48.00 | 3.24 |
| Young et al, (1987) B | 0.19 | 0.01 | 0.00 | 0.16 | 0.22 | <0.01 | 14.27 | 3.19 |
| Young et al, (1987) C | 0.21 | 0.00 | 0.00 | 0.21 | 0.21 | <0.01 | 109.67 | 3.24 |
| Young et al, (1987) D | 0.29 | 0.01 | 0.00 | 0.27 | 0.31 | <0.01 | 23.55 | 3.19 |
| **Random effects model** | 0.01 | 0.02 | 0.00 | -0.02 | 0.05 | 0.45 | 0.76 |  |
