# Supplementary material for: The effects of environmental hypoxia on substrate utilisation during exercise: a meta-analysis
Source: J Int Soc Sports Nutr. 2019 Feb 27;16:10. doi: 10.1186/s12970-019-0277-8 (PMC6391781; doi:10.1186/s12970-019-0277-8)
Supplement: Supplementary file 4 — Individual study statistics for studies evaluating relative fat oxidation during exercise matched for relative intensities in hypoxia compared with normoxia. A and B refer to the different trial arms of each study. Details of which are provided in Table 2. (DOCX 15 kb) [file 12970_2019_277_MOESM4_ESM.docx]

**Additional file 4. Individual study statistics for studies evaluating relative fat oxidation during exercise matched for relative intensities in hypoxia compared with normoxia. A and B refer to the different trial arms of each study. Details of which are provided in table 2.**

| Study | Mean difference | Standard error | Variance | Lower 95% confidence interval | Upper 95% confidence interval | p-value | Z | Weight |
| --- | --- | --- | --- | --- | --- | --- | --- | --- |
| Lundby et al, (2002) A | -1.60 | 0.45 | 0.20 | -2.48 | -0.72 | <0.01 | -3.57 | 15.47 |
| Lundby et al, (2002) B | -0.50 | 0.43 | 0.19 | -1.35 | 0.35 | 0.25 | -1.15 | 15.48 |
| Matu et al, (2017) A | 7.70 | 3.38 | 11.41 | 1.08 | 14.32 | 0.02 | 2.28 | 13.37 |
| Matu et al, (2017) B | 12.00 | 3.57 | 12.74 | 5.00 | 19.00 | <0.01 | 3.36 | 13.16 |
| Morishima et al, (2014) A | -33.70 | 2.13 | 4.54 | -37.87 | -29.53 | <0.01 | -15.82 | 14.59 |
| O’Hara et al, (2017) B | 24.30 | 4.15 | 17.20 | 16.17 | 32.43 | <0.01 | 5.86 | 12.49 |
| Peronnet et al, (2006) | -13.90 | 0.58 | 0.34 | -15.04 | -12.76 | <0.01 | -24.00 | 15.44 |
| **Random effects model** | -1.74 | 3.32 | 11.02 | -8.25 | 4.76 | 0.60 | -0.53 |  |
