# Supplementary material for: The effects of environmental hypoxia on substrate utilisation during exercise: a meta-analysis
Source: J Int Soc Sports Nutr. 2019 Feb 27;16:10. doi: 10.1186/s12970-019-0277-8 (PMC6391781; doi:10.1186/s12970-019-0277-8)
Supplement: Supplementary file 5 — Individual study statistics for studies evaluating absolute carbohydrate oxidation during exercise matched for relative intensities in hypoxia compared with normoxia. A and B refer to the different trial arms of each study. Details of which are provided in Table 2. (DOCX 15 kb) [file 12970_2019_277_MOESM5_ESM.docx]

**Additional file 5. Individual study statistics for studies evaluating absolute carbohydrate oxidation during exercise matched for relative intensities in hypoxia compared with normoxia. A and B refer to the different trial arms of each study. Details of which are provided in table 2.**

| Study | Mean difference | Standard error | Variance | Lower 95% confidence interval | Upper 95% confidence interval | p-value | Z | Weight |
| --- | --- | --- | --- | --- | --- | --- | --- | --- |
| Braun et al, (2000) | -0.73 | 0.02 | 0.00 | -0.77 | -0.69 | <0.01 | -35.51 | 15.93 |
| Lundby et al, (2002) A | -0.30 | 0.05 | 0.00 | -0.40 | -0.20 | <0.01 | -5.72 | 15.17 |
| Lundby et al, (2002) B | -0.30 | 0.07 | 0.00 | -0.43 | -0.17 | <0.01 | -4.55 | 14.69 |
| Matu et al, (2017) A | -0.38 | 0.08 | 0.01 | -0.53 | -0.23 | <0.01 | -4.92 | 14.23 |
| Matu et al, (2017) B | -0.69 | 0.08 | 0.01 | -0.85 | -0.53 | <0.01 | -8.55 | 14.08 |
| O’Hara et al, (2017) B | -1.17 | 0.16 | 0.03 | -1.48 | -0.86 | <0.01 | -7.39 | 10.41 |
| Peronnet et al, (2006) | -0.58 | 0.04 | 0.00 | -0.66 | -0.50 | <0.01 | -13.90 | 15.49 |
| **Random effects model** | -0.57 | 0.09 | 0.01 | -0.74 | -0.40 | <0.01 | -6.61 |  |
