# Supplementary material for: The effects of environmental hypoxia on substrate utilisation during exercise: a meta-analysis
Source: J Int Soc Sports Nutr. 2019 Feb 27;16:10. doi: 10.1186/s12970-019-0277-8 (PMC6391781; doi:10.1186/s12970-019-0277-8)
Supplement: Supplementary file 6 — Individual study statistics for studies evaluating absolute fat oxidation during exercise matched for relative intensities in hypoxia compared with normoxia. A and B refer to the different trial arms of each study. Details of which are provided in Table 2. (DOCX 15 kb) [file 12970_2019_277_MOESM6_ESM.docx]

**Additional file 6. Individual study statistics for studies evaluating absolute fat oxidation during exercise matched for relative intensity in hypoxia compared with normoxia.** **A and B refer to the different trial arms of each study. Details of which are provided in table 2.**

| Study | Mean difference | Standard error | Variance | Lower 95% confidence interval | Upper 95% confidence interval | p-value | Z | Weight |
| --- | --- | --- | --- | --- | --- | --- | --- | --- |
| Lundby et al, (2002) A | -0.10 | 0.00 | 0.00 | -0.11 | -0.09 | <0.01 | -21.32 | 18.10 |
| Lundby et al, (2002) B | 0.00 | 0.00 | 0.00 | -0.01 | 0.01 | 1.00 | 0.00 | 18.10 |
| Matu et al, (2017) A | 0.03 | 0.04 | 0.00 | -0.04 | 0.10 | 0.40 | 0.84 | 15.90 |
| Matu et al, (2017) B | -0.03 | 0.03 | 0.00 | -0.09 | 0.03 | 0.36 | -0.91 | 16.18 |
| O’Hara et al, (2017) A | 0.25 | 0.05 | 0.00 | 0.15 | 0.35 | <0.01 | 4.81 | 13.95 |
| Peronnet et al, (2006) | -0.27 | 0.01 | 0.00 | -0.30 | -0.24 | <0.01 | -19.38 | 17.76 |
| **Random effects model** | -0.03 | 0.04 | 0.00 | -0.11 | 0.05 | 0.44 | -0.77 |  |
