# Supplementary figures and images for: The effects of environmental hypoxia on substrate utilisation during exercise: a meta-analysis
Source: J Int Soc Sports Nutr. 2019 Feb 27;16:10. doi: 10.1186/s12970-019-0277-8 (PMC6391781; doi:10.1186/s12970-019-0277-8)

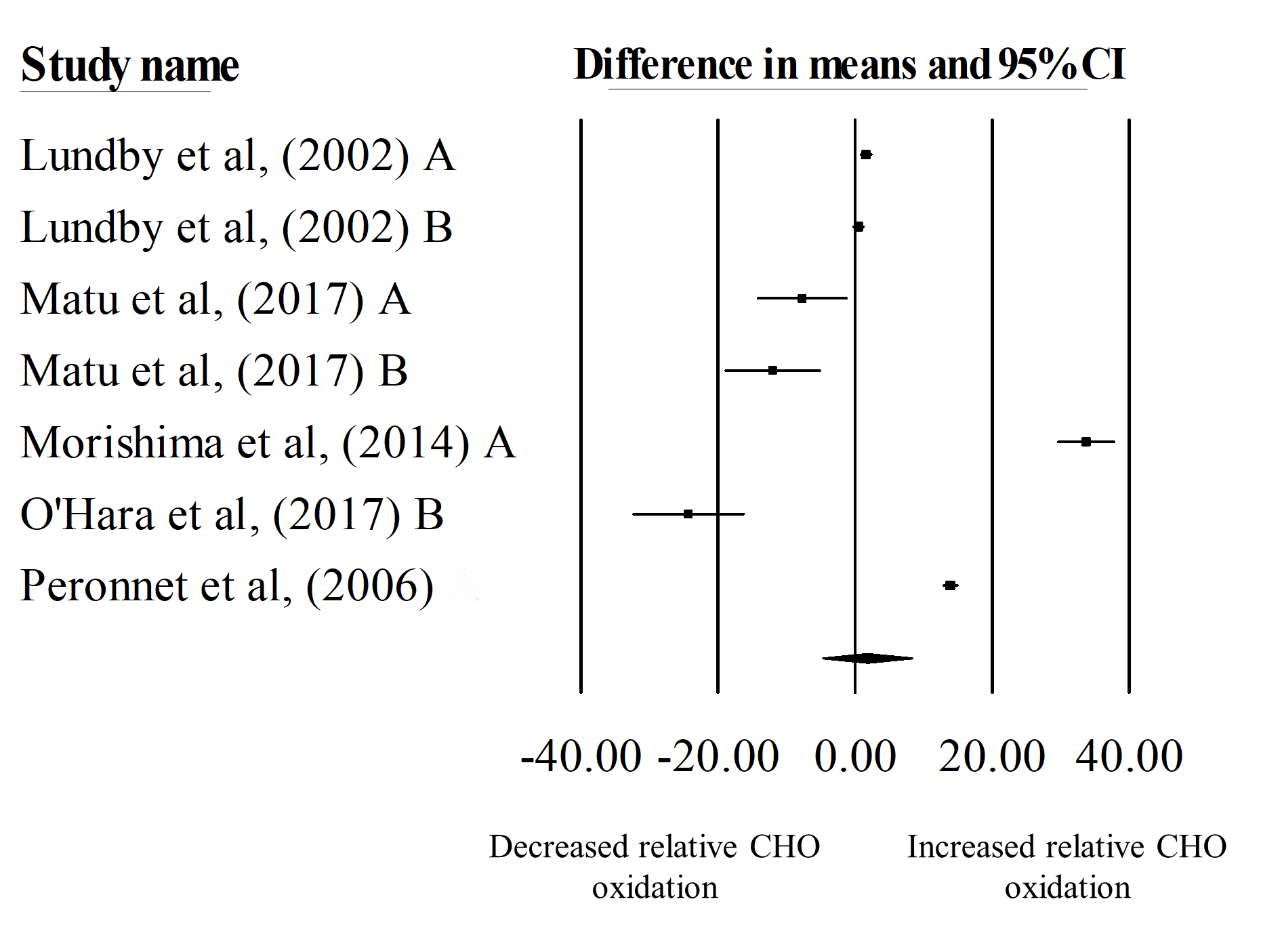

Supplement: Supplementary file 7 — Forest plot of mean differences (means ± 95% CI) for studies investigating relative carbohydrate oxidation during exercise matched for relative intensities in hypoxia compared with normoxia. The size of the circle represents the relative weight of the trial. CIs are represented by a horizontal line through their representative circles. The diamond quantifies the overall mean difference (means ± 95% CI). A and B refer to the different trial arms of each study. Details of which are provided in Table 2. (TIF 381 kb) [file 12970_2019_277_MOESM7_ESM.tif]

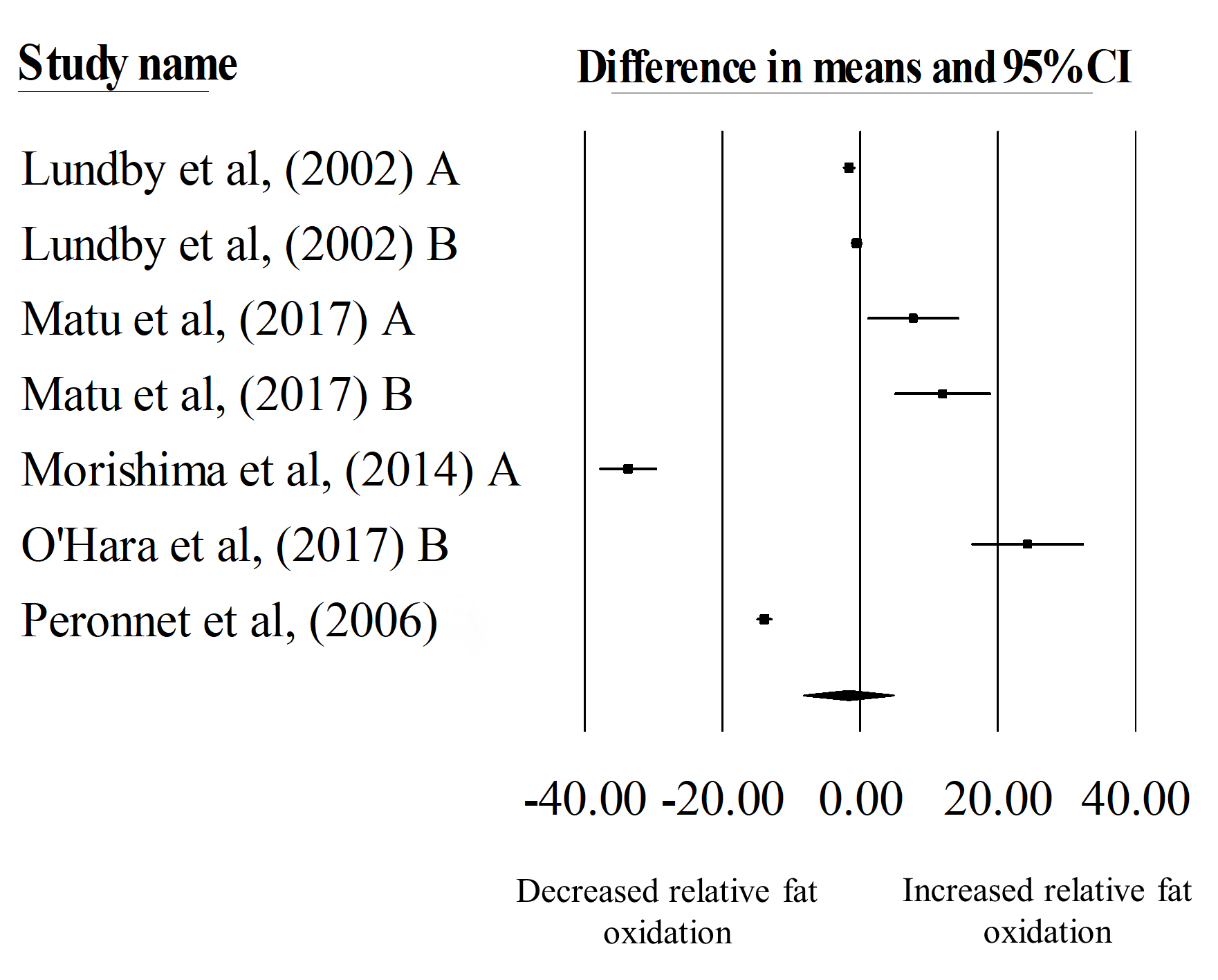

Supplement: Supplementary file 8 — Forest plot of mean differences (means ± 95% CI) for studies investigating relative fat oxidation during exercise matched for relative intensities in hypoxia compared with normoxia. The size of the circle represents the relative weight of the trial. CIs are represented by a horizontal line through their representative circles. The diamond quantifies the overall mean difference (means ± 95% CI). A and B refer to the different trial arms of each study. Details of which are provided in Table 2. (TIF 436 kb) [file 12970_2019_277_MOESM8_ESM.tif]
